# Phenotyping in *Arabidopsis* and Crops—Are We Addressing the Same Traits? A Case Study in Tomato

**DOI:** 10.3390/genes11091011

**Published:** 2020-08-27

**Authors:** Paolo Korwin Krukowski, Jan Ellenberger, Simone Röhlen-Schmittgen, Andrea Schubert, Francesca Cardinale

**Affiliations:** 1 Plant Stress Lab, Department of Agriculture, Forestry and Food Sciences DISAFA-Turin University, 10095 Grugliasco, Italy; andrea.schubert@unito.it (A.S.); francesca.cardinale@unito.it (F.C.); 2 INRES Horticultural Sciences, University of Bonn, 53121 Bonn, Germany; s.schmittgen@uni-bonn.de

**Keywords:** *Arabidopsis*, tomato, phenotyping, drought stress, translational phenotyping, osmotic stress, Dehydration, *Arabidopsis thaliana*, *Solanum lycopersicum*, *Lycopersicon esculentum*

## Abstract

The convenient model *Arabidopsis thaliana* has allowed tremendous advances in plant genetics and physiology, in spite of only being a weed. It has also unveiled the main molecular networks governing, among others, abiotic stress responses. Through the use of the latest genomic tools, *Arabidopsis* research is nowadays being translated to agronomically interesting crop models such as tomato, but at a lagging pace. Knowledge transfer has been hindered by invariable differences in plant architecture and behaviour, as well as the divergent direct objectives of research in *Arabidopsis* vs. crops compromise transferability. In this sense, phenotype translation is still a very complex matter. Here, we point out the challenges of “translational phenotyping” in the case study of drought stress phenotyping in *Arabidopsis* and tomato. After briefly defining and describing drought stress and survival strategies, we compare drought stress protocols and phenotyping techniques most commonly used in the two species, and discuss their potential to gain insights, which are truly transferable between species. This review is intended to be a starting point for discussion about translational phenotyping approaches among plant scientists, and provides a useful compendium of methods and techniques used in modern phenotyping for this specific plant pair as a case study.

## 1. Introduction

The quest for drought resistant genotypes has been, for a long time now, one of the principal challenges in plant sciences: Drought stress can seriously hamper crop development leading to a decrease in yield, with serious socio-economic consequences [1]. Historically, a decrease in crop yield has always resulted in social disorders, for example, in Egypt when the Nile flooded under emperor Claudius govern [2]; in Ireland, during the potato blight famine [3], and now seen in the effects of climate change on agriculture, including drought have been recognized, among other interconnected social, political and economic factors, as a concurring cause of the current African migration [4].

Climate change influence on temperature and rainfall occurrence and intensity is rapidly mutating the water balance of ecosystems, resulting, amidst other extreme climatic phenomena, in unusually extended drought periods in temperate countries [5]. Consequently, unless serious countermeasures are adopted, these countries may face a tremendous water shortage affecting both water and food security. According to a recent Food and Agriculture Organization (FAO) report [6], agriculture accounts nowadays for 70 per cent of water usage worldwide. It is clear that reducing its consumption in this sector could be very proficient. Such a complex task must be addressed through the combination of several integrated solutions among which the development of water-use efficient crops may hold a position of high relevance.

In the beginning, new drought resilient plants were obtained by conventional breeding among promising genotypes, exploiting the genetic pools offered by natural variation [7]. Following the advances of genetics, new methods were developed to overcome the limits of traditional breeding, attaining the possibility of gene editing at single-base definition [8].

No matter the techniques used, modified plants need to be phenotyped. Following the classical equation, where “phenotype = genotype × environment”, the mutation of a single gene can have various effects on plant phenotype [9]. *Arabidopsis thaliana* (*Arabidopsis*) has been for decades the most important model for genetics and molecular biology of angiosperms due to numerous characteristics that made it very convenient for research [10]. A short life cycle, compact dimensions, high number of seeds and a very small, sequenced and well-annotated genome. All these advantages, however, do not really overcome the fact that there is no commercial use for this weed. Consequently, *Arabidopsis* research is only a first step towards the characterization of a gene that can be useful for crop improvement. The results must be translated into more economically interesting models, such as a tomato. *Solanum lycopersicum* L. is a convenient crop model; popular for its taste and nutritional value of its fruits, it is one of the most economically important crops around the globe [11] and a high quality sequence of its genome is available [12]. Tomato is a good model for molecular, physiological and agronomical studies, and a perfect endpoint for translational biology. As an example, many tomato genes that strongly influence yield, a trait that is often overlooked in *Arabidopsis* research, are homologs of *Arabidopsis* genes involved in flowering, seed production or other reproductive processes [13]. In general, translational biology is currently undertaking the quest for adapting *Arabidopsis* molecular models to more agronomically interesting crop models, especially through the use of “omic” techniques and data mining [14]. While, possibilities and issues of *Arabidopsis*-to-crop genomic translation have been discussed elsewhere [14,15,16,17], the problematics of translating phenotyping studies have not been addressed until now. Despite both being widely used models in physiology, the different nature of *Arabidopsis* and crops prohibits an absolute equalizing of phenotyping methods and leads to different endpoints. Additionally, certain physiological variables and fruit-related traits are easier to quantify in tomato. This leads to the paradox that physiological phenotypes, described in model crops, would profit from the molecular underpinnings being investigated in *Arabidopsis*. While, meaningful physiological phenotyping of the latter plant, which is needed to correctly identify mutants in a forward genetic approach, can be a bottleneck. We believe that a careful assessment of available techniques in either plant species may help the homogenization of phenotyping methods and protocols where possible, and ease the tricky task of comparing them meaningfully. This review is a first attempt to describe the difficulties of translational phenotyping. Such a complex topic is too broad to be dissected in a single paper. Here, we will focus on translating drought stress studies from *Arabidopsis* to tomato as a case study. Drought is one of the most detrimental stressors in crop production and, as a consequence, resistance is one of the most studied traits in crop science. However, there is not a unique definition of drought and different ways to impose drought are used in experimental procedures. When comparing *Arabidopsis* and tomato studies, it is therefore important to understand the nature of drought. For instance, the drought stress that occurs during a field study in tomato differs dramatically from an osmotic stress often imposed in vitro in *Arabidopsis*.

## 2. The Multiple Facets of Drought

Drought is generally defined as a prolonged period of water shortage, resulting in an insufficient supply for the environment. However, drought stress and its precise definition, heavily rely on a number of environmental variables, as previously discussed [18], including the severity and duration of water deprivation, seasonal variations as well as the dynamics of drought occurrence, such as slightly reduced, merely suboptimal water availability or a more serious and persistent water shortage that may reveal lethal.

In plant physiology more specifically, drought is a form of stress, i.e., an external factor that seriously affects plant growth, productivity, reproductive capacity or survival [19]. As a consequence of stress, plants acclimate through a complex set of physiological, molecular, biochemical and developmental mechanisms to create a new homeostatic equilibrium. Therefore, drought can be described as water deficiency imposed (in various forms e.g., pulsed or persistent drought periods) to induce, identify and understand morphological, physiological and molecular mechanisms of acclimation [20]. Similarly, in agronomical sciences, drought is also defined in function of the studied trait. However, due to the different nature of agronomy itself, other socio-economic and environmental factors are taken into account as well. Indeed, the points of view of researchers in different scientific disciplines interested in the topic often differ noticeably. While, a molecular scientist may design a very controlled osmotic stress, in vitro, to follow the precise expression kinetic of a gene set, an agronomist may be more interested in running a field experiment to quantify whole crop stands’ yield of two genotypes, in order to identify the more tolerant one. Phenotyping performed by the two researchers will, thus, address very different traits. The type and intensity of drought stress imposed cannot be the same in both trials. Actually, the nature of the experiments the two scientists are designing and conducting will differ greatly, but plant science as a whole should still seek for ways to integrate results of both trials.

A crucial step towards understanding drought impacts across species and environments is to understand adaptation and acclimation mechanisms, and to incorporate them into experimental design.

## 3. How Do Plants Cope with Drought? A Trait-Oriented Perspective

When a drought spell occurs, plants react to raise their survival chances. There is no unique response for all plants, even when limiting the case study to *Arabidopsis* spp., responses may change dramatically among ecotypes [21]. Therefore, comparing drought stress coping strategies among different species is a complex, but a necessary task. In fact, drought acclimation strategies should be the main drivers of drought stress experiments [22].

The classical definition divides survival mechanisms in three broad categories: Drought escape, avoidance and tolerance [20,23]. In case of water scarcity, escaping plants will try to complete their life cycle before stress becomes too severe to manage (i.e., by early flowering or early maturity). In contrast, avoiding drought involves the ability of plants to maintain a stable water status despite a water shortage in soil. This is usually achieved through root architecture and water use optimization. Finally, tolerant plants will acclimate to the new environmental equilibrium and spend resources to; (a) maintain turgor in unfriendly conditions through osmotic adjustments; and (b) produce antioxidants to avoid oxidative damage caused by the generation of reactive oxygen species (ROS) as a consequence of stress. However, no plant applies only one of the three strategies. In fact, each species adopts its own combination of some drought avoidance, tolerance and escape mechanisms. This is a critical concept when comparing two different species like tomato and *Arabidopsis*.

Recently, Gilbert and Medina [22] proposed a new set of four terms linking increasing drought severity to distinct physiological mechanisms underlying the acclimation: Soil water deficit avoidance (e.g., by better soil exploration, water conservation), stress avoidance (e.g., by osmotic adjustments, optimization root-soil interactions), damage avoidance (e.g., by optimised leaf orientation, increased evaporative cooling, more favourable root-to-shoot ratio) and damage tolerance (e.g., by night-time recovery, or molecular protection conferred by heat shock proteins). Since these definitions point to the combination of specific traits and stress severity levels, they can be monitored by precise molecular and morpho-physiological markers and thus make it easier to design experiments to study preferred traits.

While tomato and *Arabidopsis* do not react in the exact same way to the same stress, they share molecular and physiological responses that are activated in response to stresses. As a consequence, we propose that in order to generate comparable datasets across species under drought, ensuring that a specific reaction of interest—be it molecular or morpho-physiological—is present at a similar level in the two species under even dissimilar environments may be more useful operationally than struggling to precisely impose the same stress to the two species. For example, in order to build a deficit irrigation protocol for tomato and potato, Jensen and colleagues [24] decided to use ABA xylem concentration to observe and synchronize stress among different species. In this way, they developed two slightly divergent watering regimes that yielded similar responses in the two *Solanaceae*. In this sense, drought stress protocols are in function of the studied traits, rather than the opposite: A similar approach is advisable when translating from *Arabidopsis* to crop and vice versa.

## 4. Drought Stress Protocols

When trying to study a drought response, scientists have to design a stress protocol suitable to follow that specific response or trait. Gilbert and Medina [22] previously discussed general experimental procedures to study different categories of responses. Instead of repeating their excellent work, we will describe which stress application methods are commonly used in both, or either plant species, discussing advantages, pitfalls and suitability for cross-species phenotyping. These protocols are often the result of a compromise between field and experimental conditions and range from very artificial in vitro setups, commonly used for molecular studies because of the absence of contamination and ease of standardisation, to open-field trials suitable for applied agricultural research (summarised in Table 1). As a general rule, the more a protocol is close to field conditions, the less its results are predictable and reproducible. When precise kinetics are to be followed (e.g., ABA accumulation in tissues, metabolite or protein accumulation, gene expression), artificial setups under very controlled conditions are more convenient.

Soil-based protocols, ranging from pot-grown plants in growth chambers or greenhouses [28] to field studies [25], are the most used when phenotyping drought stress in tomato. Their similarity to real conditions makes them perfect for applied research. Similarly, *Arabidopsis* is often grown in soil in small pots or pellets [40], while usually there is no point in studying it in the field. Drought occurs from water withdrawal in test plants, while controls are watered regularly to prevent stress responses. In general, the most obvious procedure to monitor and control stress levels is to weigh pots daily and to add different water volumes to each pot, in order to reach the same soil water content for all replicates [41]. Nonetheless, with a large experimental population such apparently trivial steps can become very time- and labour-consuming, unless a complex (and costly) automated irrigation system is available. As a consequence, do-it-yourself devices based on open source technologies, such as Arduino chipsets and/or single-board computers, are gaining interest thanks to their high versatility and cost effectiveness [31,42].

Almost all phenotyping methods discussed in this review can fit in soil-based protocols, but sometimes soil is not the recommended substrate. For example, soil dehydration is achieved through water evaporation and plant transpiration, two factors only partially controlled by the operators. Soil dehydration rates can be different among genetically identical biological replicates under identical environmental conditions, thus, reproducibility and predictability of these experiments are not always guaranteed [43]. The fact that synchronizing stress among individuals can be tricky adds complexity to this picture, especially when comparing mutants featuring differences in biomass, leaf area and/or stomatal density/width. A common, elegant solution used to minimize the latter problem is to grow mutants and wild type *Arabidopsis* plants in the same wide pot, to expose different genotypes to the same environment, better synchronizing stress appearance across individuals [44]. However, this approach may fail in comparing individuals with very different developmental features (e.g., very different root length/structure, growth rate or exudates production) and is possible only on small plants. For bigger plants phenotyping, an easy and cheap method was adopted by Marchin and colleagues [45] through a very simple hydraulic setup. The authors were able to equalise soil moisture among individuals of different species. Another concern relates to stress duration, and depending on environmental conditions, it may be controlled, only in part. Soil drying rates can be either too fast or too slow to phenotype a specific trait optimally. For example, a stress occurring too quickly can be an issue when studying late responses, such as the accumulation of osmolytes or cell wall hardening [46], or when very detailed time-courses of stress responses are to be compared between genotypes with subtle phenotypic differences. A solution can be too air-tight and cover the soil surface to lower evaporation rates. By contrast, a stress too slow to occur be concerning when very fast stress is needed to highlight differences in genotype performances, or (for example) when repeated stress is under study. In these cases, fast stress can be achieved by limiting the size of pots. In *Arabidopsis* studies, the use of peat pellets allows to achieve faster soil dehydration than in soil-filled pots, with very comparable results [26,40,47]. Surely, this is not always possible in plants, such as tomato. In this case, inert materials, such as perlite, vermiculite or rockwool are worth considering as growth substrates. These protocols are based on hydroponic-like systems where plants are grown in an inert substrate and a nutrient solution is supplied periodically [30,38]. Stress can be imposed by water withdrawal faster than soil based protocols and, if a very fast stress is needed, plants can be easily uprooted and dehydrated in air or transferred to a dry substrate [37,38]. However, care should be taken when designing fast, severe stress quickly followed by rewatering, since late responses may not have the time to be activated. Moreover, these artificial substrates lack nutrients and, consequently, nutrient stress could occur coupled with dehydration.

Sometimes, the need for a fast, precise and reproducible stress pushes researchers away from field-like conditions. While sacrificing stress authenticity, an induced physiological drought represents a good proxy of drought stress effects and allows fast and easy screening procedures; of course, it must be noted that osmotic stress slightly differs from drought stress both, at the molecular and physiological level, so care should be taken when interpreting results. Osmotic stress can be obtained supplementing growth media with osmolytes causing a decrease in the water potential of the substrate, to the point that water absorption by the plant is impaired [35,43,46,48]. While, in the past a wide range of solutes has been used, it turned out that most of them are able to penetrate plant cells resulting in a range of off-target effects dependent on the solute nature [49,50]. Therefore, the use of high molecular weight, bio-inactive compounds, such as PEG-8000 is now the standard for these experiments. Stress can be imposed to a severe degree immediately, or by gradually increasing the supplemented osmolytes and better mimicking, this way, real-world drought occurrence [43].

Systems based on PEG-infused agar are very interesting for *Arabidopsis* drought stress screenings; practically, plants can be germinated directly in PEG-infused agar or transferred at a later stage. The main reason to adopt such methods relies on their simplicity. With few manipulations, it is possible to achieve a wide range of water potentials avoiding most of the problems related to the lack of full control on environmental conditions or soil drying rates [48]. However, the same simplicity sets these models far apart from field experiments and, while it is possible, though uncommon, to adapt protocols to every stage of *Arabidopsis* growth [32], the same cannot be said of bigger plants [46]. Indeed, this approach is rarely reported on tomato, with very few examples [33]. In contrast, hydroponic systems can be easily applied to both *Arabidopsis* and tomato [34,35,36], but with potential pitfalls, for example, PEG solutions are highly viscous and can hamper aeration of the root apparatus [43]. If side effects are not a concern, other solutes, such as sorbitol or mannitol can be used. Alternatively, osmotic stress protocols can be applied to plants grown in inert substrates, obtaining a hydroponic-like system without the need for a complex apparatus [37].

When obtaining field-like conditions is not necessary, and a very fast, cost-effective and easy to handle stress is needed, dehydration can be achieved through air drying. Uprooted plants can quickly reach a severe level of stress (usually in 60–120 min), maintaining easiness of handling and independence from environmental conditions; if plants must recover from drought, it is sufficient to immerse roots in water or nutrient solution [51,52,53]. However, there are clear drawbacks: these protocols are far from field conditions and make many relevant physiological measurements difficult to carry out. Still, they can be very interesting if correctly used, as done by Fromm and colleagues when studying stomatal responses to recurring drought spells [39,54,55]. These experiments were translated to corn and rice using the same air-drying protocol [55,56], but never in tomato.

## 5. Drought Stress Phenotyping

Plant phenotyping is an incredibly broad and fast evolving research field in the plant sciences (for a recent systematic review on past development and upcoming trends in the research area, see [57]). Many excellent reviews address certain areas of plant phenotyping, ranging from the phenotyping of submicroscopic features in specific plant organs by electron microscopy, to whole plant or field of plants in agronomic contexts by UAVs (unmanned aerial vehicles) [58] and satellites. Phenotyping is often performed in specific phenotyping platforms that allow the analysis of multiple plant features at once [59] (e.g., hyperspectral reflectance, thermal signature and chlorophyll fluorescence). These platforms are particularly useful in drought stress phenotyping, as the plant environment can be precisely monitored and potentially manipulated [60]. The large costs involved in building and maintaining such platforms [61] is one limitation, along with the need for specialized personnel.

To address the challenges in translational phenotyping, we present a selection of standard drought stress phenotyping approaches in *Arabidopsis* and tomato, summarized in Table 2, and highlight similarities and differences between those approaches when applied to either species. As there are no studies directly comparing the phenotypes of *Arabidopsis* and tomato lines, there is no literature available to directly compare threshold values for single traits/quantifiable variables. Some parameters like plant height are inevitably different across species, but this does not necessarily apply to properties of the photosynthetic apparatus, or stomatal regulation. The absence of universal drought stress and phenotyping protocols, to date, still limits easy comparisons of obtained phenotypic results across species. Some examples for specific phenotyping techniques are given in the respective paragraphs.

### 5.1. Leaf Turgor Drop

Reduced leaf turgor pressure and subsequent wilting are among the first signs of drought stress, and therefore, assessed in numerous studies in both, *Arabidopsis* and tomato. In *Arabidopsis*, wilting is often not assessed as a quantitative but rather as a qualitative trait, and scientists categorize a plant as either wilted or not wilted based on visual assessment (e.g., [21]). In crops, Red Green Blue (RGB) cameras are often used to quantify projected leaf areas (reviewed e.g., in [103]), and the ratio of projected leaf area and actual leaf area can be used as an indicator of wilting. In tomato, a portable Light Detection and Ranging (LiDAR) system has been used to detect leaf angles, among other parameters [85]. Such a system, combined with powerful algorithms, can be a more useful tool than RGB images only, as more traits that are relevant for plant breeding (e.g., the dynamics of light harvesting as a function of plant architecture and daily growth rates) can be extracted from the generated point-clouds [86]. In theory, the same phenotypic methods could be used to analyze both *Arabidopsis* and tomato, as the systems are precise enough to detect changes in relatively small *Arabidopsis* leaves [104].

Whether the more detailed and more complicated phenotyping approach, described above, will replace the common practice of visual binary categorisation of *Arabidopsis* in “wilted” and “non-wilted” plants is hard to tell.

Leaf turgor can also be used to monitor plant recovery from drought stress, since during this phase, leaf water potential rises to pre-stress levels; this parameter, measured with the Scholander pressure bomb, was successfully used to monitor stress in tomato plants [38]. In *Arabidopsis* studies, the Scholander pressure bomb is rarely, used mostly due to the small dimension of the leaves, and therefore, the destructive measure of leaf Relative Water Content (%RWC) is used instead. This procedure can also monitor recovery in *Arabidopsis*, since recovered leaves have similar %RWC levels compared to pre-stress values [55,101]. Another approach to address leaf turgor is via high-precision pressure probes [62]. These systems are capable of non-destructively monitoring leaf turgor, and thereby allow insights in its temporal development under drought and during recovery. The system was, e.g., used in *Arabidopsis*, to study leaf turgor responses to several abiotic stressors, in wild-type and different mutants [63], and can replace destructive methods involving the Scholander pressure bomb.

### 5.2. Osmolarity

A key plant strategy to avoid physiological drought is to increase osmolarity within cells, leading to a more negative water potential, and therefore, an influx of water from the surrounding substrate into the plant. A standard method of destructive phenotyping is to measure the overall osmolarity of cell sap with osmometers, as done in *Arabidopsis* [32,69] and tomato [68].

Among the several classes of osmolytes (i.e., osmoprotective compounds, including sugars and amino acids), proline is the metabolite that is most commonly quantified in drought stress studies [65,66,105,106]. A recent study in tomato has suggested that the ratio of proline content in stressed and non-stressed plants can serve as an indicator for drought stress tolerance in a given genotype, with a high ratio (e.g., 1.86-fold increase in stress compared to the control) associated with the most tolerant [65]. An earlier study suggested the opposite [106], a cultivar labelled as drought stress tolerant showed no differences in leaf proline content between “stressed” and non-stressed plants. However, the reported leaf relative water content of this cultivar did not differ between treatments, suggesting that no physiological drought stress had occurred after all for otherwise undefined reasons. In *Arabidopsis*, a study highlights that proline plays a key role in the ROS scavenging system of the plant, and at the same time, acts as an osmolyte [107].

Polyamines also play a protective role against drought stress consequences, as shown in several studies in *Arabidopsis* [108,109] and tomato [110,111], at least partially by reducing ROS in the plant tissues.

The published methods to quantify leaf proline and polyamine contents are similar for *Arabidopsis* and tomato, and in theory, the same (destructive) protocols could be used. If similar drought stress protocols are applied, it may be feasible to transfer knowledge on drought resistance from *Arabidopsis* to tomato, based on osmolyte accumulation patterns as a readout.

### 5.3. Water Loss at the Leaf Level

Both direct and indirect analyses of stomatal dynamics can be conducted in *Arabidopsis* and tomato in similar ways. For the rather direct analysis via (microscopic) images of the leaves, a fixation of the tissue is performed, which can be done by creating a die with nail polish [112] or by fixating leaves using the chemical glutaraldehyde [55]. Stomata can subsequently be counted and measured under an optical or confocal microscope. For more sophisticated analyses, variable pressure scanning electron microscopes are used to address stomata features [29]. Using this method, a fixation of leaf material is not necessary and damage through fixation can be avoided. Recent advancements in automated image analysis will probably pave the way to an automated analysis of relevant stomatal features like density, length, width and guard cell size from microscopic images [113].

The analysis of trichomes in drought studies is common, as these specialized epidermal cells manipulate the microclimate of the thin air layer surrounding the leaf, and can thereby, prevent unproductive water losses. Enhanced trichome density in drought tolerant genotypes is found in tomato [29] and *Arabidopsis* [114], and can be assessed via light microscopy or scanning electron microscopy.

A common, non-invasive, although indirect, method in addressing transpiration is thermal imaging. This technique has been used to identify *Arabidopsis* mutants defective in stomatal regulation already in 2002 [70]. The combination of thermal and visible images was later used to remotely access drought stress in crops under greenhouse and field conditions. Sunlit and shaded leaves were separated using RGB-image data and the corrected thermal information correlated fairly well with measured stomatal conductance [71].

Stomatal conductance—and thereby transpiration through stomata—can also be assessed using a Porometer, as previously described in *Arabidopsis* [72] and tomato [38,73]. Devices measuring carbon assimilation can also provide information on leaf transpiration, with more precision than the latter instrument but with longer measurement times.

Whole-plant transpiration dynamics are observed with gravimetric systems. In short, potted plants are placed on wages and the growth substrate is covered by water-impermeable materials to avoid evaporation. This also allows for a calculation of water use efficiency (WUE) in its agronomic sense as either biomass or yield produced per unit of transpired water. Efforts are being made to combine 3D imaging systems (capable of estimating biomass) with gravimetric transpiration control, allowing dynamic phenotyping over time [115]. A commercially available gravimetric system has been used in tomato already, addressing drought stress tolerance of an introgression population [28].

Stomatal water loss is also used to analyze recovery when a plant is re-watered after stress, stomata start reopening and gas exchange rates reach values very close to pre-stress ones. However, it is important to note that stomatal conductance does not fully recover immediately after stress, as it does not depend only on hydraulic signals. Therefore, even when leaf water potential or %RWC are back to the levels of irrigated plants, stomatal conductance will lag behind (hysteresis of stomata closure). This phenomenon, often called “after effect” of drought, is well documented both in *Arabidopsis* and tomato [38,55,116] and it is by all means a reflection of drought stress memory at the stomatal level [116].

### 5.4. Gas Exchange

Gas exchange and carbon assimilation measurements are straightforward ways to assess the photosynthetic efficiency of a plant in a given environment. A drop in gas exchange can be a sign of a range of different plant stresses, including drought. In *Arabidopsis*, LI-COR gas exchange systems were used in several studies to assess leaf gas exchange under drought [117,118]. In tomato, carbon assimilation under drought stress is studied across different scales and levels of environmental control, from chambers with artificially elevated CO_2_ [100] to greenhouse and field [25,26]. As carbon assimilation is highly influenced by irradiation and temperature, studies in greenhouses and in the field should be conducted in reproducible weather conditions, ideally during sunny days and virtually at the same time. For studies in the field, hand-held devices are the most practical choice. Good care has to be taken when comparing leaf gas exchange values across studies: a study on tomato [26] reports 0.15–0.25 µmol H_2_O m^−2^ s^−1^, with slight differences between control and drought, while a study on *Arabidopsis* [99] reports a more than four-fold increase during drought stress, but still lower absolute values of stomatal conductance than any tested tomato (0.02–0.09 µmol H_2_O m^−2^ s^−1^). As drought stress protocols, instrument settings (e.g., photon flux density) and growth systems are inconsistent across studies, the comparison of absolute carbon assimilation rates across studies (and species) is inappropriate.

Carbon fluxes inside the plant can be studied in even more detail by using ^13^CO_2_ and mass spectrometry [98].

### 5.5. Enhanced Chlorophyll Fluorescence

As drought stress impairs photosynthetic activity and enhanced chlorophyll fluorescence is a direct result of this impairment [119], the quantification of chlorophyll fluorescence is a standard procedure in stress phenotyping both in *Arabidopsis* and horticultural crops [119,120]. In general, a plant that maintains high photochemical quenching, and therefore relatively low non-photochemical quenching and associated variable chlorophyll fluorescence under stress conditions, is described as tolerant against this stressor. In tomato, imaging systems are mainly used in molecular studies on plants in early growth stages and in artificial environments like growth chambers (e.g., [77]), while at later growth stages, and/or in less artificial environments like greenhouses, leaf clip-based systems are more commonly used (e.g., [121]). However, it is possible to apply fluorescence imaging in commercial-like greenhouses [75]. Many chlorophyll fluorescence measurement systems require a dark adaptation of measured leaves; a prerequisite that may be hard to fulfil, depending on the growth system.

### 5.6. ROS and Leaf Secondary Metabolite Contents

The formation of ROS is a hallmark of cellular stress also upon drought; it can be observed in vivo, based on the oxidation of fluorescence probes like H2DCFDA, as shown in *Arabidopsis* [78]. In the presence of ROS, this chemical starts to emit fluorescence signals that can be observed with hyperspectral cameras. While destructive assessment of ROS is carried out in tomato (e.g., [79,122]), the recently introduced method of non-destructive, whole-plant ROS imaging is to our knowledge not yet applied in tomato, despite the potential for knowledge transfer on ROS production and scavenging mechanisms.

A common measure to address persistent stress is the quantification of secondary metabolites (SM) with the capability to reflect or absorb excessive amounts of sunlight, thus, mitigating the risk of excessive ROS production, and also to scavenge ROS directly [123,124]. SMs such as flavonoids or anthocyanins can be quantified destructively, as done in *Arabidopsis* [125] and tomato [126]. Identification and quantification of SMs can be achieved photometrically (e.g., [127]), via High Performance Liquid Chromatography (HPLC) (e.g., [128]) or via Gas Chromatography-Mass spectrometry (GC-MS) (e.g., [117]). The latter allows a more precise analysis of chemical subgroups of metabolites, potentially offering detailed insights in their metabolism (“metabolomics”). When the researcher is interested in the spatial or temporal development of SM contents, the use of either imaging [81,83] or non-imaging [111,112] remote sensors should be considered to avoid destructive measurements. Several non-imaging sensors rely on leaf clipping, and therefore, require a minimum leaf size, which can be a limiting factor especially in *Arabidopsis*. For reviews on available devices, see [104,113]. Many hyperspectral imaging systems can be used not only under lab conditions, but are also extensively used in the field, as they are, either hand-held [114] or can be mounted on UAVs for rapid phenotyping of large numbers of plants [58]. Factors like leaf age and morphology may have a large impact on SMs estimation based on non-destructive methods [115], and therefore must be taken into account.

### 5.7. Root Structure

Roots can either be phenotyped two-dimensionally, by using a normal camera and plants grown either hydroponically or in agar (e.g., [88,90]); or three-dimensionally for plants grown in systems closer to actual crop production systems (e.g., [91]). While the former are quick, easy and cheap, the latter allows more sophisticated analyses of complex traits like three-dimensional (3D) root system architecture (RSA).

RSA phenotyping allows dynamic interactions between roots and their surrounding substrate to be understood by evaluating, e.g., fine root diameters, specific root length, root angles and root length density (reviewed by [129]). Understanding genotypic differences in RSA responses to abiotic stressors, like drought has the potential to improve the breeding of resilient cultivars [130,131]. In order to analyze dynamic rhizosphere interactions and spatial alterations, recommended detection methods do not interfere with the ‘natural’ habitat of roots [132]. Particular approaches mostly refer to plants grown artificially in hydroponics, paper pouches, gel and in appropriate soil types, inter alia in soil-filled rhizotrons (up to a volume of ~18 L), which limits phenotyping to young or small plants [131]. Growth media limitations do also apply for 3D methods, like magnetic resonance imaging [130] and X-ray [133], visualizing the ‘natural’ growth and architecture, as well as the impacts of biotic and abiotic stresses. In order to bridge the gap between phenotype and genotype, recent studies revealed insight into intertwined genetic factors of root and shoot development, in both, *Arabidopsis* and Solanum [89,134]. However, plants are often analyzed during their early growth and transferability to mature plants may be limited [135].

### 5.8. Changes in Vegetative Growth

Leaf area densities and related source-sink relationships are known to be important for final yield in horticultural crops [127] and grains. These traits are therefore studied extensively in crops, but the *Arabidopsis* model is due to its compact habitus unsuitable for translation of most information in this respect. The differences in growth habitus between *Arabidopsis* and tomato indeed complicate a homogenization of phenotyping methods regarding vegetative growth. While the rosette-like structure of *Arabidopsis* allows relatively straightforward analyses, the three-dimensional structure of tomato is more difficult to parametrize. For tomato indeed, not only leaf area index (LAI), but also leaf area density (LAD) in several horizontal layers within a high-wire-system tomato canopy have been analyzed with the LiDAR-based system described above [60]. In *Arabidopsis* instead, 3D plant architecture analyses are not common, as its rosette-like structure is rather plain. So, the additional information on the third dimension does not seem to justify the effort needed to capture it, and stress effects can be detected as projected leaf area observed non-destructively via RGB cameras located above the plants [59].

### 5.9. Changes in Generative Growth

Early fruit set is also part of the drought escape strategy and therefore a symptom of drought stress both in *Arabidopsis* [136] and tomato [100]. Many genes that apparently control yield in tomato, especially through the regulation of auxin contents, are homologs of genes found in *Arabidopsis* [13]. However, there are major differences in generative growth of the two model plants. Tomato is a plant insensitive to daylength, e.g., the fruit set is not influenced by season [137], whereas *Arabidopsis* flowers earlier under long-day conditions [138]. Thus, researchers interested in drought-induced early flowering in *Arabidopsis* and tomato have to take day length (in-) sensitivity of the respective plant into account, either through appropriate experimental design and/or through statistical models.

Fruit yield is a highly integrative phenotypic trait, and genetically controlled by at least 28 QTLs in tomato [139]. Operationally, the temporal development of generative growth can be assessed quite easily, as flowers and fruit setting are directly visible in both *Arabidopsis* [140] and tomato. Direct yield quantification in tomato is common, although quite labor intensive, as fruits must be harvested once a week over a period of several weeks to obtain robust results. Also, to obtain meaningful results, plants must be grown in commercial-like systems, an often challenging task for molecular biology groups.

Another important difference in reproductive physiology of *Arabidopsis* and tomato that has to be considered is that the short life cycle in the former ends with fruit production, whereas constant fruit production over months and theoretically over years is possible with indeterminate tomato varieties.

### 5.10. Observing Stress through Marker Genes

After sensing drought, plants start activating a complex network of gene-expression changes affecting plant behaviour. While some of these may vary among plant species, others are pretty well-conserved, thus, representing a signature of drought stress. Transcripts of such marker genes are often quantified in physiological studies and can be used to monitor stress response intensities.

Describing the specific intricacies of molecular responses during drought stress, a complex and still partially elusive network, is far from the purposes of this review; among the impressive body of literature on the topic, the reader is referred to two up to date and influential reviews [141,142]. Here, we will quickly suggest some useful stress marker genes that are shared (or not) between the two species.

Some of the most prominent molecular responses to drought stress are governed by the stress hormone ABA (abscisic acid). Firstly, ABA biosynthesis is augmented during stress through the transcriptional induction of the genes encoding its biosynthetic enzymes. Among these, the *NCED* (*9-Cis-Epoxycarotenoid Deoxygenase*) genes, which catalyze one of the last steps of ABA biosynthesis, can be used to monitor plant sensing of drought stress in tomato and *Arabidopsis*. *AtNCED3* is expressed quickly during drought stress [94] as soon as *Arabidopsis* leaves lose turgor [97]. In tomato, the two genes *SlNCED1* and *SlNCED2* seem to play similar roles [95,96]. ABA-responsive genes can be used as stress markers, too: the transcript of the dehydrin-encoding gene *AtRD29B* (*Responsive to Dehydration 29 B*) is typically profiled in drought stress experiments [39,99] and possesses a similarly behaving orthologue in tomato: *SlRD29* [100].

Another commonly used drought stress marker gene in *Arabidopsis* is *Homeobox Protein 6* (*HB6*), an ABA-activated gene in drought stress that encodes a transcription factor governing several stress responses [40,101]; however, no obvious tomato homologue has been characterized until now. Similarly, the tomato ABA-dependent, dehydrin-encoding *Solyc02g084850* is a good drought marker (our unpublished data) still uncharacterized in *Arabidopsis*.

In some cases, such as the study of genotypes with disturbed ABA sensing/biosynthesis, the use of ABA-dependent stress markers may not be appropriate. In this case, ABA-independent, drought-activated genes can be used instead; one of these is *DREB2* (*Dehydration-responsive Element-Binding protein 2*). Both *AtDREB2A* and *SlDREB2* expression is induced in either plant species by drought stress [40,98,99,102], and they encode for ABA-independent transcription factors, involved in drought stress responses; signalling genes downstream of *DREB2* are, consequently, good putative stress markers as well.

## 6. Conclusions

Nowadays, more than 200 angiosperm species have been sequenced, and this number is predicted to increase rapidly [143]. Together with the levels reached by our understanding of genetics, this is raising consistently the possibility of developing new marketable crop genotypes suitable for future agricultural challenges. However, until these new genotypes are characterized, they remain just a possibility: the need for precise phenotyping is stronger than ever. In spite of the difficulties outlined in the introduction, some efforts in adjusting drought stress and phenotyping protocols across species have already been made, and technological advances in plant phenotyping offer further potential for translational phenotyping. Therefore, we hope that future research efforts will account for the need of comparable phenotyping in *Arabidopsis* and crops.

As technology evolves, phenotyping facilities addressing multiple traits simultaneously are becoming the new standard in plant phenotyping [120,144]. The combination of several of the techniques mentioned above allows integrated phenotyping to a detail level never matched before, and that could never be reached by single-sensor approaches. As the often mentioned phenotyping bottleneck [145] is gradually being overcome, the scientific focus will have to shift towards developing universal phenotyping approaches which integrate results of phenotypic observations across scales, environments, and even across species. In this sense, the advent of phenomics [146] coupled with the newest bioinformatic approaches such as machine learning [147] will probably play a major role in this transition. Still, more traditional phenotyping approaches will always be necessary to some extent.

The knowledge gathered on the *Arabidopsis* model is more valuable than ever, especially if the scientific community manages to translate it to crop models from which we can obtain a real advantage, including in food, fodder or fibre. We are convinced that knowledge can be better translated between species in relation to mechanisms involved in tolerance against abiotic stresses like drought, as well as on many other plant traits, such as fruit development, light response, or resistance against pests and diseases. At present, the transferability of knowledge is still limited, as stress protocols, as well as phenotyping protocols (if at all existent) are often incoherent among different species. Researchers interested in translating the vast knowledge gained on *Arabidopsis* to crops and vice versa must carefully design their studies and ideally build interdisciplinary teams to gather knowledge on genetic background, expected and desired phenotypes and on the agricultural production systems the crops are grown in.

While the idea of modelling the performance of plants with virtual allele combinations under a range of environments is not new [148], it seems that its potential has still not been realized, to date. Some of the existing molecular and physiological plant models of water status and drought stress in tomato (e.g., [149,150]) and *Arabidopsis* (e.g., [151]) may be connected to improve our understanding of drought and plant responses to it. Moreover, new modelling approaches, including the causal inference approaches by Pearl and colleagues, which provide mathematical tools to describe causal relations, rather than correlation, and explicitly include the scientist’s causal knowledge in the design of a statistical model. These methods, until now widely overlooked in the plant sciences, have the potential to allow insights in systems hardly comparable by classic statistical approaches [152], and may thereby help to lift translational phenotyping to the next level.

## Figures and Tables

**Table 1:** Drought stress protocols commonly used in *Arabidopsis* and/or tomato. The table discriminates protocols based on the stress application method; for each protocol, growth substrates, advantages and disadvantages, phenotyping suitabilities are listed. When possible, an example for both plants is given.

Stress Application Method	Growth Substrate	Advantages (+)/Disadvantages (−)	Phenotyping Suitability	*Arabidopsis*	Tomato
Water withholding	Soil (open or protected field)	(+) realistic drought conditions	All traits can be phenotyped, but root phenotyping can be unfeasible	NA	Landi et al., 2017 [25]
		(+) best method for market-oriented phenotyping			
		(−) other stresses such as salinity and heat can co-occur			
		(−) not used/useful for *Arabidopsis*			
		(−) strongly affected by weather conditions			
	Soil (pot)	(+) quite close to commercial conditions	All phenotyping methods here described can be used, but root phenotyping needs appropriate apparatus (e.g., rhizotrons, x-ray tomography)	Vello et al., 2015 [26]	Visentin et al., 2016 [27] Halperin et al., 2017 [28] Galdon-Armero et al., 2018 [29]
		(+) suitable for every growth stage			
		(−) influenced by environmental conditions			
		(−) can be laborious			
		(−) stress can be slow to occur			
	Soil (pellet)	(+) as for pot protocols, but the limited size of pellets speeds up drought stress occurrence	All phenotyping methods described here can be used	Vello et al., 2015 [26]	NA
		(−) not used for tomato			
	Inert substrate e.g., sand, vermiculite (pot)	(+) stress is reached faster than in soil-based protocols	All phenotyping techniques described here can be carried out	Santaniello et al., 2017 [30]	Takayama et al., 2011 [31]
		(+) easier to uproot plants			
		(−) nutrient stress occurs together with water withholding, as plants are fertigated			
		(−) more artificial than soil-based protocols			
Transfer to stressing substrate	Agar with low osmotic potential	(+) very reproducible	Phenotyping, especially for tomato, is limited to the first stages of plant growth (seedling stage). Very convenient for early screenings	Frolov et al., 2017 [32]	Aazami et al., 2010 [33]
		(+) a wide range of stress intensities can be achieved			
		(+) fast			
		(+) sterile			
		(−) far from naturally occurring conditions			
		(−) depending on osmolyte nature, off-target effects can be a concern			
		(−) suitable only for small/young plants			
		(−) stomata dynamics hard to assess in very young plants			
	Hydroponics-Osmotic stress	(+) very reproducible	All phenotyping techniques described here can be carried out. Very suitable for the description of precise kinetics. Absence of soil makes root phenotyping not always feasible	Nieves-Cordones et al., 2012 [34]	Ali et al., 2019 [35] Amitai-Ziegerson et al., 1995 [36]
		(+) fast			
		(+) a wide range of stress intensities can be achieved by gradually increasing osmolyte concentration			
		(−) artificial			
		(−) depending on solute nature, off-target effects can be a concern			
		(−) root growth is altered			
		(−) need for a hydroponic apparatus			
	Inert substrates-Osmotic stress	(+) reproducible	All phenotyping techniques described here can be carried out. Very good if precise kinetics are analyzed.	NA	Jin et al., 2000 [37]
		(+) fast			
		(+) a wide range of stress intensities can be achieved by gradually increasing osmolyte concentration			
		(+) cost-effective			
		(−) artificial			
		(−) depending on solute nature, off-target effects can be a concern			
Transfer to dry substrate	Inert substrate	(+) very fast	Due to very fast stress, only early responses can be studied. Root phenotyping is not convenient	NA	Visentin et al., 2020 [38]
		(+) reproducible			
		(−) very artificial			
		(−) severe stress only			
		(−) only early responses can be analyzed			
Uproot and let dehydrate	Inert substrate to no substrate	(+) very fast	Due to very fast stress, only early responses can be studied. Root phenotyping is not convenient	Virlouvet et al., 2014 [39]	NA
		(+) reproducible			
		(−) very artificial			
		(−) severe stress only			
		(−) only early responses can be analyzed			

**Table 2:** An overview of common phenotyping targets in *Arabidopsis* and tomato under drought. Referenced publications contain detailed information on the methods applied.

Physiological Reaction Monitored	Accessible Traits	*Arabidopsis*	Tomato
Leaf turgor drop	- Direct assessment (high-precision pressure probe)	Direct assessment:	Direct assessment: Lee et al., 2012 [62]
	- Wilting (RGB-imaging)	Ache et al., 2010 [63]	
	- Drop in projected leaf area		Plant architecture (Light Detection and Ranging—LiDAR):
	- Lower specific leaf area	Wilting (RGB-imaging): Bouzid et al., 2019 [21]	Rose et al., 2015 [64]
	- Relative water content	Projected leaf area:	
		de Ollas et al., 2019 [47]	
Osmolarity increase	- proline quantification	Proline:	Proline: Aghaie et al., 2018 [65]
	- osmolarity quantification	Li et al., 2019 [66]	Osmolarity:
		Zhang et al., 2013 [67]	Rodríguez-Ortega et al., 2019 [68]
		Osmolarity:	
		Frolov et al., 2017 [32]	
		Versluis & Bray, 2004 [69]	
Stomata closure	- Leaf temperature (by infrared thermography)	Infrared thermography:	Infrared
	- Direct stomata aperture measurements (by microscopy; destructive)	Li et al., 2017 [44]	thermography:
	- Stomatal conductance (by porometer)	Merlot et al., 2002 [70]	Leinonen & Jones, 2004 [71]
		Kuromori et al., 2011 [72]	Porometer:
		Microscopy:	Visentin et al., 2020 [38]
		Virlouvet & Fromm, 2014 [55]	Caird et al., 2007 [73]
			Microscopy:
			Galdon-Armero et al., 2018 [29]
Lower carbon fixation	- Leaf gas exchange	Harb et al., 2010 [40]	Galdon-Armero et al., 2018 [29]
Enhanced chlorophyll fluorescence	- Hand-held devices to assess chlorophyll fluorescence	Hand-held device:	Imaging system (within crop stand):
	- Fluorescence imaging (e.g., PAM imaging)	Jung, 2004 [74]	Takayama et al., 2011 [75]
		PAM imaging:	Imaging system:
		Yao et al., 2018 [76]	(FluorCamFC1000-H)
			Mishra et al., 2012 [77]
Higher concentrations of Reactive Oxygen Species (ROS) in the leaf	- Chemical staining and imaging: destructive or non destructive	Non-destructive chemical imaging:	Destructive chemical imaging:
		Fichman et al., 2019 [78]	Ijaz et al., 2017 [79]
		Destructive chemical imaging:	
		Lee et al., 2012 [80]	
Higher concentrations of ROS-scavenging secondary metabolites (e.g., flavonoids, anthocyanins, carotenoids)	- Hand-held devices for accessing specific leaf compounds (e.g., Dualex, Multiplex, FieldSpec)	Hyperspectral imaging:	Hyperspectral imaging: Susič et al., 2018 [81]
	- Hyperspectral imaging	Mishra et al., 2019 [82]	Metabolomics: Ali et al., 2018 [35]
	- Full metabolic profiling (destructive)	Matsuda et al., 2012 [83]	
		Metabolomics:	
		Nakabayashi et al., 2014 [84]	
Changes in vegetative growth	- RGB-Imaging: lower projected leaf area, compact habitus	RGB-Imaging:	LiDAR: Hosoi et al., 2011 [85]
	- Lower fresh and dry mass	Ollas et al., 2019 [47]	3D point clouds: Paulus et al., 2014 [86]
	- Lower specific leaf area	Senescence: Jin et al., 2018 [87]	Trichomes: Galdon-Armero et al., 2018 [29]
	- Slowed longitudinal growth of individual leaves		
	- Senescence		
Changes in root growth	- 2D features	Xu et al., 2013 [88]	Alaguero-Cordovilla et al., 2018 [89]
	- 3D features	Mathieu et al., 2015 [90]	Mairhofer et al., 2012 [91]
Changes in generative growth	- Earlier fruit set	Seed mass and yield: Jofuku et al., 2005 [92]	Flowering and yield: Sivakumar et al., 2016 [93]
	- Lower fruit weight		
	- Higher number of non-marketable fruits		
	- Lower overall yield		
Molecular markers	*- 9-Cis-Epoxycarotenoid Deoxygenase*	*AtNCED3:*	*SlNCED1, SlNCED2:*
	*NCED*	Hao et al., 2009 [94]	Yu et al., 2019 [95]
		Sussmilch, 2017	Muoz-Espinoza et al., 2015 [96]
		[97]	
			*SlRD29:*
	*- Responsive to dehydration 29*	*AtRD29B:*	Gao et al., 2020 [98]
	*RD29*	Ma et al., 2019 [99]	Iovieno et al., 2016 [100]
		Virlouvet et al., 2014 [39]	
			NA
	*- Homeobox protein 6*	*HB6:*	
	*HB6*	Ding et al., 2013 [101]	
		Harb et al., 2010 [40]	
	*- Solyc02g084850*		(Unpublished data)
		NA	
	*- Dehydration-responsive Element- Binding protein 2*		*SlDREB2:*
	*DREB2*	*AtDREB2A:*	Gao et al., 2020 [98]
		Ma et al., 2019 [99]	Hichri et al., 2016 [102]
		Harb et al., 2010 [40]	
